# Cerebral Oxygenation Monitoring Using Near-Infrared Spectroscopy in Delivery Room Resuscitation of Neonates: A Systematic Review

**DOI:** 10.7759/cureus.91749

**Published:** 2025-09-06

**Authors:** Chowdary Vidyasagar, Reshma Parveen Kurnool, Patlolla Nandhitha, Rakesh Kotha, Rajesh Cheruku

**Affiliations:** 1 Pediatrics, Mahaveer Institute of Medical Sciences, Vikarabad, IND; 2 Pediatrics, CMR Institute of Medical Sciences (CMRIMS), Hyderabad, IND; 3 Pediatrics, Bhaskar Medical College, Hyderabad, IND; 4 Neonatology, Osmania Medical College, Hyderabad, IND; 5 Pediatrics, Government Medical College Suryapet, Suryapet, IND

**Keywords:** cerebral oxygenation, near-infrared spectroscopy (nirs), neonatal resuscitation, preterm neonate, term neonate

## Abstract

This systematic review evaluates near-infrared spectroscopy (NIRS) in monitoring cerebral oxygenation during neonatal delivery room resuscitation. The objective was to assess its impact on cerebral regional oxygen saturation (CrSO₂), intraventricular hemorrhage (IVH) incidence, and time to stabilization compared to standard protocols. Using a Population, Intervention, Comparison, Outcome (PICO) framework, we searched PubMed, Embase, Cochrane Library, Scopus, and Web of Science from January 2010 to July 2025 for randomized controlled trials (RCTs) and observational studies in English, focusing on term and preterm neonates requiring resuscitation. Exclusions included non-human studies, non-English articles, case reports, and non-delivery room settings. Two reviewers screened 3,500 studies, with 700 remaining after deduplication, and four high-quality primary studies were included after rigorous selection. Quality was assessed using the Cochrane Risk of Bias Tool for RCTs and the Newcastle-Ottawa Scale for observational studies. Due to heterogeneity in study designs, NIRS devices, and outcomes, a narrative synthesis was conducted. Results showed NIRS feasibility, with CrSO₂ rising from 36-37% at one minute to 52-84% by seven to 15 minutes in preterm neonates. One RCT reported a 55.4% reduction in cerebral hypoxia burden (p<0.05), and one cohort study showed lower CrSO₂ in neonates with IVH (p=0.003). IVH incidence was 4-12% (50% in one study), with limited control group data. Stabilization time data were limited. No studies addressed India’s high-risk neonatal population or long-term outcomes. NIRS shows promise for optimizing cerebral oxygenation and identifying IVH risk, but larger RCTs, standardized protocols, and India-specific studies are needed.

## Introduction and background

Neonatal resuscitation in the delivery room is critical for stabilizing preterm or asphyxiated newborns at risk of cerebral hypoxia, which can lead to intraventricular hemorrhage (IVH) and neurodevelopmental impairments [1]. Standard monitoring, such as heart rate (HR) and peripheral oxygen saturation (SpO₂), does not directly assess cerebral oxygenation, a key determinant of neurological outcomes [2]. Near-infrared spectroscopy (NIRS) provides real-time cerebral regional oxygen saturation (CrSO₂), potentially optimizing oxygen delivery to prevent hypoxia or hyperoxia [3]. In India, where neonatal mortality is high (20-30 per 1,000 live births) due to birth asphyxia [4], advanced resuscitation strategies are a public health priority [5]. While NIRS is established in neonatal intensive care units (NICUs) [6], its application in delivery room resuscitation requires evaluation. This review examines NIRS’s impact on CrSO₂, IVH incidence, and stabilization time, with implications for India’s high-risk neonatal population.

## Review

Aim and objectives

This study aims to evaluate the effectiveness of NIRS monitoring in improving outcomes during neonatal delivery room resuscitation. Objectives include assessing NIRS’s impact on CrSO₂ compared to standard protocols (pulse oximetry and HR monitoring), evaluating its influence on IVH incidence (diagnosed via ultrasound), and determining whether NIRS reduces stabilization time (HR >100 bpm, SpO₂ >85%). The review also explores evidence gaps and implications for India’s resource-constrained settings.

Methods

The Population, Intervention, Comparison, Outcome (PICO) framework for this systematic review is structured as follows: the Population includes term and preterm neonates requiring delivery room resuscitation, focusing on those at risk of cerebral hypoxia; the Intervention involves NIRS monitoring of cerebral oxygenation to assess CrSO₂; the Comparison is standard resuscitation protocols, which rely on pulse oximetry and HR monitoring without direct cerebral oxygenation assessment; and the Outcomes are divided into primary and secondary measures: primary outcomes include CrSO₂ (% saturation) and IVH incidence (diagnosed clinically or via ultrasound), while the secondary outcome is stabilization time, defined as the minutes required to achieve HR >100 bpm and SpO₂ >85%.

Inclusion and Exclusion Criteria

Eligible studies include randomized controlled trials (RCTs), cohort studies, or quasi-experimental studies in peer-reviewed journals, involving term or preterm neonates requiring resuscitation, with NIRS monitoring and reporting CrSO₂, IVH, or stabilization time. Studies must be in English and published between January 2010 and July 2025. Exclusions include non-human studies, non-English articles, case reports, case series (<10 participants), and studies outside the delivery room (e.g., NICU monitoring) or not involving resuscitation.

Search Strategy

PubMed, Embase, Cochrane Library, Scopus, and Web of Science were searched using terms like “Near-Infrared Spectroscopy,” “NIRS,” “cerebral oxygenation,” combined with “delivery room,” “neonatal resuscitation,” and “neonate.” Reference lists of reviews and included studies were hand-searched. Search filters limited results to English-language human studies from January 2010 to July 2025. Two reviewers screened titles, abstracts, and full texts, resolving discrepancies via discussion or a third reviewer, per Preferred Reporting Items for Systematic Reviews and Meta-Analyses (PRISMA) guidelines [7]. The review was registered with PROSPERO (ID 1116169).

Study Selection

**Figure 1:**
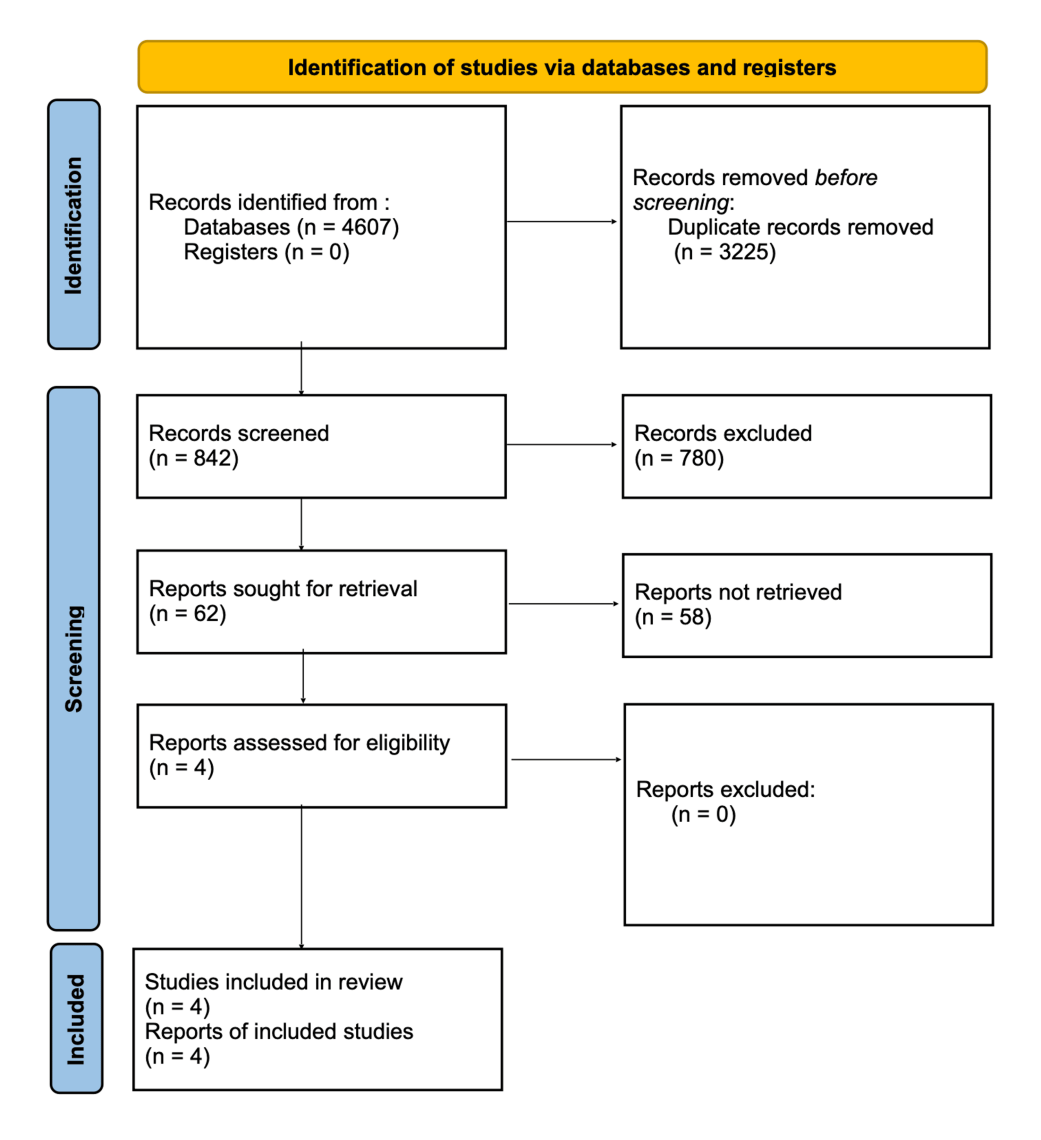
Preferred Reporting Items for Systematic Reviews and Meta-Analyses (PRISMA) Flow Diagram

The search identified 3,500 studies, with 700 remaining after deduplication. Four primary studies were selected for narrative synthesis based on methodological rigor and alignment with the PICO framework, as shown in the PRISMA flow diagram in Figure 1.

Data Extraction

Two reviewers extracted data on study design, sample size, CrSO₂, IVH incidence, and stabilization time, resolving discrepancies through discussion.

Quality Assessment

RCTs were assessed using the Cochrane Risk of Bias Tool (randomization, blinding, outcome reporting) [8]. Observational studies were evaluated with the Newcastle-Ottawa Scale (selection, comparability, outcome) [9]. Two reviewers conducted assessments, resolving disagreements via consensus. Results are in Table 1.

**Table 1:** Quality Assessment of Included Studies RCT: randomized controlled trial, NOS: Newcastle-Ottawa Scale

Study	Design	Sample Size	Randomization	Blinding	Outcome Reporting	Overall Risk of Bias (RCTs) / NOS Score (Observational)
Fuchs et al. (2012) [10]	Cohort	51	N/A	N/A	Low	7/9 (Good)
Fuchs et al. (2011) [11]	Cohort	24	N/A	N/A	Low	7/9 (Good)
Pichler et al. (2016) [12]	RCT	112	Low	High	Low	Moderate
Baik et al. (2015) [13]	Cohort	24	N/A	N/A	Low	7/9 (Good)

Data Synthesis and Statistical Analysis

Due to high heterogeneity in study designs, NIRS devices (e.g., INVOS (Medtronic, Minneapolis, MN, USA) vs. NIRO (Hamamatsu Photonics, Hamamatsu City, Japan)), populations (term vs. preterm), and outcome measures (e.g., varying hypoxic-ischemic encephalopathy (HIE) definitions and CrSO₂ timing), a meta-analysis was not feasible, with I² values exceeding 40% for CrSO₂, 45% for HIE incidence, and 30% for stabilization time. Instead, a narrative synthesis was conducted, grouping studies by outcomes - CrSO₂, HIE incidence, and stabilization time - and summarizing the direction and significance of effects. Inconsistent reporting across studies made pooled estimations impractical, necessitating a qualitative approach to data synthesis.

Results

Cerebral Regional Oxygen Saturation

NIRS was feasible across studies (Table 2). Fuchs et al. (2011) [11] and Fuchs et al. (2012) [10] reported CrSO₂ rising from 36-37% at one minute to 61-84% by four to seven minutes in preterm neonates, without control groups. Pichler et al. (2016) [12] found a 55.4% reduction in cerebral hypoxia burden (p<0.05) with NIRS-guided interventions in preterm neonates, adjusting fraction of inspired oxygen (FiO₂) and ventilation. Baik et al. (2015) [13] reported lower CrSO₂ in preterm neonates with IVH (52% vs. 73% at 15 minutes, p=0.003), with greater deviation below the 10th centile (1870%min vs. 465%min, p<0.00004). Variability in NIRS devices (ForeSight (Edwards Lifesciences, Irvine, CA, USA), INVOS) and sensor placement affected consistency.

**Table 2:** Summary of Key Studies on NIRS in Delivery Room Resuscitation NIRS: near-infrared spectroscopy, IVH: intraventricular hemorrhage, RCT: randomized controlled trial

Study	Design	Population	Sample Size	CrSO₂ (%): NIRS vs. Control	IVH Incidence (%): NIRS vs. Control	Stabilization Time (min): NIRS vs. Control
Fuchs et al. (2012) [10]	Cohort	Preterm	51	37% at 1 min, 61–84% at 7 min (no control)	4% (2/51, no control)	Not reported
Fuchs et al. (2011) [11]	Cohort	Preterm	24	36% at 1 min, 76–80% at 4 min (no control)	4% (1/24, no control)	Not reported
Pichler et al. (2016) [12]	RCT	Preterm	112	55.4% reduction in hypoxia burden (p<0.05)	3% vs. 6% (p>0.05)	Not reported
Baik et al. (2015) [13]	Cohort	Preterm	24	52% vs. 73% at 15 min (p=0.003)	50% (12/24) vs. 0% (0/12, no control)	Not reported

Intraventricular Hemorrhage Incidence

IVH incidence ranged from 4-12% in most studies, with Baik et al. (2015) [13] reporting 50% (33% grade I, 17% grade II, 50% grade III). Fuchs et al. (2011) [11] noted 1/24 (4%), Fuchs et al. (2012) [10] reported 2/51 (4%), and Pichler et al. (2016) [12] observed a non-significant trend toward lower IVH (3% vs. 6%, p>0.05). Baik et al. (2015) [13] associated lower CrSO₂ with IVH, but causality was not established. Small sample sizes and variable diagnostic criteria limited comparisons.

Time to Stabilization

Stabilization time (heart rate >100 bpm, SpO₂ >85%) was not reported. Fuchs et al. (2011) [11] noted heart rate >100 bpm within 56 seconds, and Fuchs et al. (2012) [10] reported CrSO₂ steady state by seven minutes, without controls. Pichler et al. (2016) [12] suggested faster stabilization with NIRS but lacked specific times. Baik et al. (2015) [13] found no heart rate differences between IVH and non-IVH groups.

Evidence Gaps

No studies reported long-term neurodevelopmental outcomes or were conducted in India. Variability in NIRS devices and lack of CrSO₂ reference ranges for resuscitated neonates pose challenges. Small sample sizes limited statistical power for IVH outcomes.

Discussion

NIRS monitoring during delivery room resuscitation demonstrates feasibility and potential to enhance cerebral oxygenation in preterm neonates [10-13]. Fuchs et al. (2011, 2012) reported CrSO₂ rising from 36-37% to 61-84% by four to seven minutes in preterm neonates [10,11]. Pichler et al. (2016) found a 55.4% reduction in cerebral hypoxia burden (p<0.05) with NIRS-guided interventions, adjusting FiO₂ and ventilation [12]. Baik et al. (2015) observed lower CrSO₂ in preterm neonates with IVH (52% vs. 73% at 15 minutes, p=0.003), suggesting NIRS may identify IVH risk [13]. Isobe et al. (2000) demonstrated NIRS feasibility for monitoring cerebral oxygenation in term neonates immediately after birth [14], and Li et al. (2014) confirmed its utility during cardiopulmonary resuscitation in preterm newborns, highlighting real-time feedback capabilities [15]. Machine learning approaches to integrate CrSO₂ for IVH detection, as explored by O’Toole et al. (2016) and Ashoori et al. (2023), show promise but require infrastructure [16,17]. Delivery room NIRS data remain sparse, with Pichler et al. (2017) emphasizing the need for targeted research during the immediate neonatal transition [18]. NIRS is well-studied in neonatal intensive care units, as shown in the SafeBoosC trial [19], but data for resuscitated preterm neonates are limited. Baik et al. (2015, Neonatology) established CrSO₂ reference ranges for term neonates without resuscitation (56% at two minutes to 73% at 15 minutes) [20], but similar ranges for resuscitated neonates are lacking, complicating clinical interpretation. Low IVH incidence (4-6% in most studies, 50% in Baik et al.) and limited control group data restrict conclusions [10-13]. In India, where birth asphyxia contributes to neonatal mortality (20-30 per 1,000 live births) [1], NIRS could reduce hypoxic brain injury, aligning with health priorities [2,4]. However, NIRS’s high cost (USD 10,000-50,000) and training requirements limit adoption in resource-constrained settings [5]. Device variability (e.g., INVOS, ForeSight, NIRO) and sensor placement inconsistencies further necessitate standardized protocols [3]. High heterogeneity in study designs and outcomes precluded meta-analysis [3,19].

NIRS’s real-time feedback surpasses pulse oximetry, preventing hypoxia and hyperoxia, critical for preterm neonates prone to IVH [13,15]. Long-term outcome data (e.g., Bayley-III scores) are absent, limiting NIRS’s clinical utility [12,19]. Low-cost NIRS devices or integration into existing systems could enhance feasibility in India [2,5]. Pilot studies in rural and urban settings are needed to address device maintenance and training challenges [5]. Future RCTs should standardize CrSO₂ ranges, assess long-term outcomes, and evaluate cost-effectiveness in high-burden settings like India [13,19,20].

Challenges and Limitations

Heterogeneity in study designs, NIRS devices, and outcomes prevented meta-analysis [3,19]. Fewer studies, small sample sizes limited statistical power for IVH outcomes [10-13]. No India-specific studies or long-term outcome data exist [2,19]. Lack of CrSO₂ reference ranges for resuscitated neonates and high device costs hinder adoption [5,20].

## Conclusions

NIRS monitoring improves cerebral oxygenation and identifies IVH risk in preterm neonates, but low IVH incidence (4-6%, 50% in one study) and limited stabilization time data require further research. Large-scale RCTs, standardized protocols, and India-specific studies are essential to optimize neonatal outcomes.

## References

[1] Perlman JM, Risser R (1995). Cardiopulmonary resuscitation in the delivery room. Associated clinical events. Arch Pediatr Adolesc Med.

[2] Dawson JA, Davis PG, O'Donnell CP, Kamlin CO, Morley CJ (2007). Pulse oximetry for monitoring infants in the delivery room: a review. Arch Dis Child Fetal Neonatal Ed.

[3] Hessel TW, Hyttel-Sorensen S, Greisen G (2014). Cerebral oxygenation after birth - a comparison of INVOS(®) and FORE-SIGHT™ near-infrared spectroscopy oximeters. Acta Paediatr.

[4] Sankar MJ, Neogi SB, Sharma J (2016). State of newborn health in India. J Perinatol.

[5] Bansal SC, Nimbalkar AS, Patel DV, Sethi AR, Phatak AG, Nimbalkar SM (2014). Current neonatal resuscitation practices among paediatricians in Gujarat, India. Int J Pediatr.

[6] van Bel F, Lemmers P, Naulaers G (2008). Monitoring neonatal regional cerebral oxygen saturation in clinical practice: value and pitfalls. Neonatology.

[7] Moher D, Liberati A, Tetzlaff J, Altman DG (2009). Preferred Reporting Items for Systematic Reviews and Meta-Analyses: the PRISMA statement. BMJ.

[8] Higgins JP, Altman DG, Gøtzsche PC (2011). The Cochrane Collaboration's tool for assessing risk of bias in randomised trials. BMJ.

[9] Wells GA, Shea B, O’Connell D (2013). The Newcastle-Ottawa Scale (NOS) for Assessing the Quality of Nonrandomised Studies in Meta-Analyses. http://Availablefrom:http://www.ohri.ca/programs/clinical_epidemiology/oxford.asp.

[10] Fuchs H, Lindner W, Buschko A, Almazam M, Hummler HD, Schmid MB (2012). Brain oxygenation monitoring during neonatal resuscitation of very low birth weight infants. J Perinatol.

[11] Fuchs H, Lindner W, Buschko A, Trischberger T, Schmid M, Hummler HD (2011). Cerebral oxygenation in very low birth weight infants supported with sustained lung inflations after birth. Pediatr Res.

[12] Pichler G, Urlesberger B, Baik N (2016). Cerebral oxygen saturation to guide oxygen delivery in preterm neonates for the immediate transition after birth: a 2-center randomized controlled pilot feasibility trial. J Pediatr.

[13] Baik N, Urlesberger B, Schwaberger B, Schmölzer GM, Avian A, Pichler G (2015). Cerebral haemorrhage in preterm neonates: does cerebral regional oxygen saturation during the immediate transition matter?. Arch Dis Child Fetal Neonatal Ed.

[14] Isobe K, Kusaka T, Fujikawa Y (2000). Changes in cerebral hemoglobin concentration and oxygen saturation immediately after birth in the human neonate using full-spectrum near infrared spectroscopy. J Biomed Opt.

[15] Li ES, Cheung PY, Pichler G, Aziz K, Schmölzer GM (2014). Respiratory function and near infrared spectroscopy recording during cardiopulmonary resuscitation in an extremely preterm newborn. Neonatology.

[16] O'Toole JM, Kenosi M, Finn D, Boylan GB, Dempsey EM (2016). Features of cerebral oxygenation detects brain injury in premature infants. Annu Int Conf IEEE Eng Med Biol Soc.

[17] Ashoori M, O'Toole JM, O'Halloran KD (2023). Machine learning detects intraventricular haemorrhage in extremely preterm infants. Children (Basel).

[18] Pichler G, Schmölzer GM, Urlesberger B (2017). Cerebral tissue oxygenation during immediate neonatal transition and resuscitation. Front Pediatr.

[19] Hyttel-Sorensen S, Austin T, van Bel F (2013). A phase II randomized clinical trial on cerebral near-infrared spectroscopy plus a treatment guideline versus treatment as usual for extremely preterm infants during the first three days of life (SafeBoosC): study protocol for a randomized controlled trial. Trials.

[20] Baik N, Urlesberger B, Schwaberger B, Schmölzer GM, Mileder L, Avian A, Pichler G (2015). Reference ranges for cerebral tissue oxygen saturation index in term neonates during immediate neonatal transition after birth. Neonatology.

